# Circulating insulin-like growth factor-I, insulin-like growth factor binding protein-3 and terminal duct lobular unit involution of the breast: a cross-sectional study of women with benign breast disease

**DOI:** 10.1186/s13058-016-0678-4

**Published:** 2016-02-18

**Authors:** Hisani N. Horne, Mark E. Sherman, Ruth M. Pfeiffer, Jonine D. Figueroa, Zeina G. Khodr, Roni T. Falk, Michael Pollak, Deesha A. Patel, Maya M. Palakal, Laura Linville, Daphne Papathomas, Berta Geller, Pamela M. Vacek, Donald L. Weaver, Rachael Chicoine, John Shepherd, Amir Pasha Mahmoudzadeh, Jeff Wang, Bo Fan, Serghei Malkov, Sally Herschorn, Stephen M. Hewitt, Louise A. Brinton, Gretchen L. Gierach

**Affiliations:** Metabolic Epidemiology Branch, Division of Cancer Epidemiology and Genetics, National Cancer Institute, National Institutes of Health, 9609 Medical Center Drive, Rm. 7-E108, Bethesda, MD 20892-9774 USA; Present Affiliation: Food and Drug Administration, Silver Spring, MD USA; Breast and Gynecologic Cancer Research Group, Division of Cancer Prevention, National Cancer Institute, National Institutes of Health, Bethesda, MD USA; Biostatistics Branch, Division of Cancer Epidemiology and Genetics, National Cancer Institute, National Institutes of Health, Bethesda, MD USA; Usher Institute of Population Health Sciences and Informatics, The University of Edinburgh, Edinburgh, Scotland; McGill University, Montreal, Québec Canada; Present Affiliation: Northwestern University Medical School, Chicago, IL USA; University of Vermont, Burlington, VT USA; Department of Radiology and Biomedical Imaging, University of California, San Francisco, San Francisco, CA USA; Present Affiliation: Hokkaido University, Graduate School of Medicine, Sapporo, Japan; Laboratory of Pathology, Center for Cancer Research, National Cancer Institute, National Institutes of Health, Bethesda, MD USA; Office of the Director, Division of Cancer Epidemiology and Genetics, National Cancer Institute, National Institutes of Health, Bethesda, MD USA

## Abstract

**Background:**

Terminal duct lobular units (TDLUs) are the primary structures from which breast cancers and their precursors arise. Decreased age-related TDLU involution and elevated mammographic density are both correlated and independently associated with increased breast cancer risk, suggesting that these characteristics of breast parenchyma might be linked to a common factor. Given data suggesting that increased circulating levels of insulin-like growth factors (IGFs) factors are related to reduced TDLU involution and increased mammographic density, we assessed these relationships using validated quantitative methods in a cross-sectional study of women with benign breast disease.

**Methods:**

Serum IGF-I, IGFBP-3 and IGF-I:IGFBP-3 molar ratios were measured in 228 women, ages 40-64, who underwent diagnostic breast biopsies yielding benign diagnoses at University of Vermont affiliated centers. Biopsies were assessed for three separate measures inversely related to TDLU involution: numbers of TDLUs per unit of tissue area (“TDLU count”), median TDLU diameter (“TDLU span”), and number of acini per TDLU (“acini count”). Regression models, stratified by menopausal status and adjusted for potential confounders, were used to assess the associations of TDLU count, median TDLU span and median acini count per TDLU with tertiles of circulating IGFs. Given that mammographic density is associated with both IGF levels and breast cancer risk, we also stratified these associations by mammographic density.

**Results:**

Higher IGF-I levels among postmenopausal women and an elevated IGF-I:IGFBP-3 ratio among all women were associated with higher TDLU counts, a marker of decreased lobular involution (*P-*trend = 0.009 and <0.0001, respectively); these associations were strongest among women with elevated mammographic density (*P*-interaction <0.01). Circulating IGF levels were not significantly associated with TDLU span or acini count per TDLU.

**Conclusions:**

These results suggest that elevated IGF levels may define a sub-group of women with high mammographic density and limited TDLU involution, two markers that have been related to increased breast cancer risk. If confirmed in prospective studies with cancer endpoints, these data may suggest that evaluation of IGF signaling and its downstream effects may have value for risk prediction and suggest strategies for breast cancer chemoprevention through inhibition of the IGF system.

**Electronic supplementary material:**

The online version of this article (doi:10.1186/s13058-016-0678-4) contains supplementary material, which is available to authorized users.

## Background

Terminal duct lobular units (TDLUs) comprise the secretory units of the breast and are the structures from which most breast cancers and their precursors arise. Microscopically, TDLUs consist of terminal ducts and substructures referred to as acini. With aging, TDLUs involute, which results in reduced acini content per TDLU (i.e., decreased TDLU epithelial sub-structures), shorter TDLU span and eventually lower TDLU numbers. Data suggest that reduced age-related TDLU involution among women with benign breast disease is a marker of increased breast cancer risk [1–5]. However, factors and mechanisms that account for the substantial observed inter-individual variation among women are not well-described [6]. There are several ways to determine the degree of lobular involution of the breast including counting the number of TDLUs or acini within a TDLU structure [6], measuring the diameter of lobules [6], or characterizing lobule type [5]. Assessing factors associated with TDLU involution, as assessed qualitatively or quantitatively, could elucidate mechanisms related to breast cancer risk and suggest strategies for risk prediction following a benign breast biopsy or preventive strategies that accelerate the involution process. The insulin-like growth factor (IGF) system represents a promising candidate pathway that may influence breast cancer risk by modulating TDLU involution.

In rodent models, the IGF system interacts with hormonal and non-hormonal pathways to influence normal breast development over time, including breast development during puberty, pregnancy, and the post-lactational involution that accompanies weaning [7–9]. In women, associations between circulating IGF-I concentrations and breast cancer risk have been robust. A recent pooled analysis of data from approximately 20,000 pre- and postmenopausal women included in 17 prospective studies concluded that elevated IGF-I was associated with increased breast cancer risk, and this association did not vary significantly by menopausal status at blood collection [10]. Nonetheless, poor reproducibility between IGF assays, small effect sizes, and weaker effects for prospective studies as compared with case-control studies have led to diverging views about the importance of the IGF system in breast cancer [11, 12].

In the Nurses’ Health Study, elevated IGF-I and a higher IGF-I:IGFBP-3 ratio were associated with decreased TDLU involution, based on visual assessment [13]. In the same study, increased IGF receptor staining in TDLUs of women with benign proliferative breast disease was associated with increased risk of subsequent breast cancer. Similarly, we hypothesize that higher serum levels of IGF-I and the IGF-I:IGFBP-3 ratio are associated with lower levels of TDLU involution among women with benign breast disease. Further, we suggest that IGFs exert their most important effects on normal breast tissue, early in the process of breast cancer development; IGFs, therefore, may influence breast cancer risk indirectly by shaping the molecular histology upon which other factors act. Thus, assessing the IGF system and its relationship to breast cancer risk may require an understanding of its effects on benign breast tissue and the effects other downstream factors may have on the at-risk epithelium of the TDLU. We hypothesize that elevated mammographic density, a clinical factor associated with both increased breast cancer risk and decreased TDLU involution of the breast [1, 14–16], may be an important complementary cofactor. Specifically, TDLU involution, as assessed histologically, provides a detailed snapshot of breast structure in a small region of the breast, whereas mammographic density represents a global measure of non-fatty tissue, but does not distinguish between epithelium and non-fatty stroma.

To assess the possible associations between the IGF system and TDLU involution, we evaluated serum levels of IGF-I, IGFBP-3 and the molar ratio of IGF-I:IGFBP-3 among women with benign breast disease in relation to three standardized, reproducible quantitative measures that are inversely related to TDLU involution [6]: numbers of TDLUs per unit of tissue area (TDLU count), the diameter of the TDLU (TDLU span), and acini counts per TDLU. Further, we assessed the potential effect modification of these associations by mammographic density.

## Methods

### Study population

The Breast Radiology Evaluation and Study of Tissues (BREAST) Stamp Project is a cross-sectional molecular epidemiologic study of mammographic density in 465 women ages 40 to 65 years, referred for diagnostic biopsy from 2007 through 2010 at the University of Vermont (UVM) College of Medicine and its affiliated academic medical center, the UVM Medical Center (formerly Fletcher Allen Health Care) [17]. The current analysis included women in the BREAST Stamp Project with available serum (>3.2 mL serum available), who were not using exogenous hormones at the time of biopsy, and who had available quantitative histologic assessment of TDLU involution, resulting in 281 eligible women for IGF-I and IGFBP-3 serum testing (see below). Of the 281 women with IGF-I and IGFBP-3 measurements, we further excluded 47 women with breast cancer and 6 without TDLU data, resulting in an analytic sample of 228 women, including 155 premenopausal and 73 postmenopausal women. For 2 of the 228 women, it was unknown whether they were current users of menopausal hormone therapy. These women were included in the analytic population, given that their exclusion did not alter the study results. Participants provided written informed consent, which included providing access to medical records and mammographic images; Institutional Review Boards at the University of Vermont and the National Cancer Institute approved this study.

### Study procedures

Women were referred for image-guided breast biopsy following an abnormal breast imaging examination. Digital mammographic examinations were performed with a density phantom, permitting measurement of percent volumetric mammographic density using single x-ray absorptiometry (SXA) [18]. For the same digital images that were selected for SXA analyses, measures of percent dense area were estimated, as described previously [19, 20], using interactive, computer-assisted thresholding software comparable to other validated methods [21]. Percent volumetric mammographic density as measured using SXA has been found to be accurate to equivalent measures acquired from magnetic resonance imaging (MRI) of the breast [22], and significantly associated with breast cancer risk [20, 23]. Density measures used in this study are from the ipsilateral breast on which the biopsy was performed and TDLU measures assessed.

Demographic and breast cancer risk factor information was collected using a self-administered questionnaire (see sample questionnaire at http://breastscreening.cancer.gov/ [24]) and a supplementary telephone interview. A woman was considered postmenopausal if menstrual periods had stopped more than 12 months prior to interview, if she had undergone bilateral oophorectomy, or if she had undergone hysterectomy (or gynecologic surgery associated with cessation of menses) and was 55 years of age or older; otherwise, a woman was considered premenopausal. Given the age range of our study population, the premenopausal subgroup likely contains a mixture of both pre- and perimenopausal women. For a subset of premenopausal women with previously measured serum follicle stimulating hormone (FSH) and estradiol levels (n = 105), we identified perimenopausal women (n = 19) as previously described [25]. Body mass index (BMI, kg/m^2^) was computed from measures of height and weight obtained on the day of the breast biopsy. Whole blood samples were collected prior to breast biopsy using standard techniques, allowed to clot for 30 minutes, and processed at the UVM General Clinical Research Center. Samples were centrifuged at 3,000 rpm for 15 minutes, and the serum and clot fractions were frozen at –80 °C until shipment to SeraCare Life Sciences (Gaithersburg, MD, USA), where they were stored in liquid nitrogen.

### Laboratory measures of IGF-I and IGFBP-3

IGF-I and IGFBP-3 levels were measured in duplicate in masked serum samples by enzyme-linked immunosorbent assay (ELISA) (Diagnostic Systems Laboratory (Webster, TX, USA)) as described previously [26]. Three quality control (QC) samples (follicular phase, luteal phase and postmenopausal samples) were measured in duplicate in a masked fashion with each of the eight assay batches performed. The mean intra-batch coefficients of variation (CVs) were 2.7 % for IGF-I and 5.1 % for IGFBP-3, and the inter-batch CVs were 10.5 % for IGF-I and 10.5 % for IGFBP-3. Mean IGF-I and IGFBP-3 levels for each subject’s duplicate measurements were used in the analyses. To approximate the circulating free, bioactive levels of IGF-I, the molar ratio of IGF-I to IGFBP-3 was estimated as previously described [27, 28]. Overall and menopausal status-specific tertiles of IGF-I, IGFBP-3 and their molar ratio were calculated and used in subsequent association analyses.

### Pathology

Ultrasound-guided core needle (14-gauge needle) or stereotactic-guided vacuum-assisted (9-gauge needle) breast biopsies were routinely processed as formalin-fixed paraffin-embedded blocks, which were sectioned and stained with hematoxylin and eosin (H&E) for diagnosis. For study purposes, final diagnoses were categorized as non-proliferative benign breast disease, proliferative (ductal hyperplasia; sclerosing adenosis), proliferative with atypia (atypical ductal or lobular hyperplasia), in-situ*,* or invasive breast carcinoma based on review of microscopic sections and surgical pathology reports. The present analysis was restricted to women whose biopsy diagnoses were benign (Additional file 1: Table S1). Digitized images of sections from the target block were used for analysis of TDLU involution (described below).

### Histologic assessment of TDLU involution

H&E-stained tissue sections were digitized at × 20 magnification (Aperio ScanScope CS, Vista, CA, USA), and prepared for web-based viewing and annotation with Digital Image Hub software (SlidePath/Leica, Dublin, Ireland). The lasso tool in Digital Image Hub was used to manually outline and measure total tissue area (mm^2^) per section. For women who had multiple benign biopsy targets, measures from the first biopsy were used (n = 5). Sections were visually assessed to estimate percentage fat (0, 1, 5, 10, 20, 30, 40, 50, 60, 70, 80, 90, 95 and 100 %). Normal TDLUs per section were enumerated, and up to 10 TDLUs were evaluated for maximum diameter as TDLU span (measured with an electronic ruler in microns) and categories of acini counts per TDLU (categories: 1, ≤10; 2, 11–20; 3, 21–30; 4, 31–50; 5, 51–100; and 6, >100) to provide reliable estimates, as previously described [6]. Median values for each woman were used as summary measures of TDLU span and acini count. Overall and menopausal status-specific tertiles of median TDLU span and median category of acini counts per TDLU were calculated and used in subsequent analyses. A previous study demonstrated high intra-observer agreement (Spearman’s *r* >0.90) for the study pathologist (MES) for the TDLU measures [6].

### Statistical analysis

Because IGF levels have been shown to differ by menopausal status [10, 29], analyses of associations between circulating IGF-I or IGFBP-3 levels and TDLU measures were done for all women combined and women stratified by menopausal status. Circulating IGF levels and TDLU measures in premenopausal and postmenopausal women were compared using the Kruskal–Wallis rank test. Relationships between circulating IGF levels, TDLU measures and mammographic density were assessed with Spearman rank correlation; Spearman partial rank correlation was also estimated after removing the effects of age and BMI. To account for excess zero TDLU counts in the study population, multivariable zero-inflated Poisson (ZIP) regression [30] was used to estimate relative risks (RRs) and 95 % confidence intervals (CIs) for the relationship between circulating IGF levels (in tertiles) and number of TDLUs (i.e., TDLU count). ZIP regression models included tissue area as an offset to account for differences in biopsy needle gauges. Potential confounders were identified separately for pre- and postmenopausal women for inclusion in the multivariable models. We also explored inclusion of interaction terms in sensitivity analysis. Among all women, interactions between BMI and menopausal status and BMI and percent fat on the tissue slide were statistically significant; however, their inclusion did not alter the observed RRs, and the most parsimoniously adjusted model is therefore presented. Among postmenopausal (but not premenopausal) women, we identified a significant interaction between BMI and percent fat on the tissue slide, which was retained in the final model to improve fit even though its inclusion did not alter results for the main exposures.

Among women with at least one TDLU, ordinal logistic regression models were used to estimate odds ratios (ORs) and 95 % CIs for associations between circulating IGF levels (independent variable, categorized into tertiles) and the outcomes of median TDLU span and median category of acini count per TDLU (categories: 1, ≤10; 2, 11–20; 3, 21–30; 4, 31–50; 5, 51–100; and 6, >100). For these analyses, adjustment factors were identified by stepwise selection with an inclusion/exclusion criterion of *P* <0.05 and *P* >0.10, respectively. The variables that were considered included age at biopsy, BMI, ages at menarche, first birth and menopause (among menopausal women), parity and percent fat on the tissue slide. Final models for each analysis are included within the respective table footnotes.

We tested for effect modification of the association between IGF levels and TDLU count by stratifying models on mammographic density (menopausal-specific tertiles of percent volumetric density). Stratified analyses were adjusted for the confounders identified in the main analysis with the exception of the interaction between BMI and percent fat on the tissue slide, as its inclusion yielded similar results, and age at menarche in the stratum of the highest mammographic density tertile among postmenopausal women, in order to improve model convergence. We used the Wald test to compare the estimates from models stratified by tertiles of mammographic density to obtain a *P* value for interaction (*P*-int). Sensitivity analyses using percent dense mammographic area yielded similar conclusions (data not shown). In sensitivity analyses, we also restricted analyses of premenopausal women to those with available hormone data, excluding potentially perimenopausal women. In addition, we mutually adjusted models relating IGF-I and IGFBP-3 to TDLU counts for IGFBP-3 and IGF-I, respectively. Statistical analyses were conducted using Stata version 11.2 (Statacorp, College Station, TX, USA), with the exception of the tests for interaction which were conducted in R version 2.14.1 [31].

## Results

### Participant characteristics

Overall, 94 % of subjects were white and 68 % were premenopausal. The median age at biopsy for premenopausal women was 47 years and for postmenopausal women was 57 years (Table 1). Approximately 74 % of premenopausal and 77 % of postmenopausal women in our study population were parous. Nearly half (45 %) of parous postmenopausal women had age at first birth <25 years, whereas only 24 % of parous premenopausal women had a first birth at <25 years of age (*P* = 0.01, Table 1). As previously reported in this study population [17], volumetric and area percent mammographic density measures were higher among premenopausal as compared with postmenopausal women (Additional file 1: Table S1).

**Table 1:** Selected characteristics of women in the BREAST Stamp Project with benign breast disease, stratified by menopausal status

Characteristic	Premenopausal: n = 155		Postmenopausal: n = 73		
	age = 40–55 (median 47) years		age = 44–64 (median 57) years		
	Number	Percent	Number	Percent	*P* ^t^
Age at biopsy (years)					
40–44	53	34.2	1^*^	1.4	<0.001
45–49	64	41.3	2	2.7	
50–54	37	23.9	21	28.8	
55–59	1	0.6	24	32.9	
60–65	0	0	25	34.2	
Race/Ethnicity					
White, non-Hispanic	147	94.8	67	91.8	0.37
Other	8	5.2	6	8.2	
Body mass index (kg/m^2^)					
<25	84	54.2	32	43.8	0.34
25.0–29.9	35	22.6	21	28.8	
≥30	36	23.2	20	27.4	
Age at menarche (years)					
≤12	56	36.1	30	41.1	0.36
13	60	38.7	21	28.8	
≥14	38	24.5	21	28.8	
Missing	1	0.6	1	1.4	
Parity					
Nulliparous	41	26.4	17	23.3	0.12
1	13	8.4	14	19.2	
2	65	41.9	29	39.7	
≥3	36	23.2	13	17.8	
Age at first birth (years)					
<25	37	23.9	33	45.2	0.01
25–29	44	28.4	14	19.2	
≥30	33	21.3	9	12.3	
Oral contraceptive use					
Never	23	14.8	10	13.7	0.82
Ever	132	85.2	63	86.3	
Type of menopause					
Natural	~	~	53	72.6	~
Surgical	~	~	3	4.1	
Type unknown	~	~	17	23.3	
Age at menopause (years)					
≤45	~	~	21	28.8	~
46–49	~	~	13	17.8	
50–52	~	~	19	26	
≥53	~	~	13	17.8	
Unknown	~	~	7	9.6	
Any menopausal hormone therapy use^a^					
Never	135	87.1	49	67.1	<0.001
Ever	19	12.3	24	32.9	
Missing	1	0.6	0	0	
Cigarette smoking (100+ cigarettes/lifetime)					
Never	83	53.5	28	38.4	0.07
Former	55	35.5	31	42.5	
Current	10	6.5	10	13.7	
Missing/Unknown	7	4.5	4	5.5	
Family history of breast cancer^b^					
0	114	73.5	52	71.2	0.96
≥1	41	26.5	19	26	
Missing	0	0	2	2.7	

^a^Data on any use of menopausal hormone therapy (MHT) missing for one premenopausal woman, and current MHT use missing for one postmenopausal woman who reported ever use. ^b^Women with at least one first degree relative with a diagnosis of breast cancer. *P* ^t^ is the value for testing for differences in distribution by menopausal status using the chi square (*Χ* ^2^) test or Fisher's exact test when the number for a category was <5. *This woman experienced natural menopause

### Relationships between IGF-I, IGFBP-3 and TDLU metrics

Median levels of IGF-I and the IGF-I:IGFBP-3 molar ratio were higher among premenopausal women as compared with postmenopausal women (*P* ≤0.001; Table 2), whereas levels of IGFBP-3 were not significantly different between these two groups (*P* = 0.44). Correlations between TDLU count, TDLU span and acini count were generally similar by menopausal status, such that TDLU span and acini count were correlated (*r* >0.60; *P* ≤0.001), whereas TDLU count was not significantly correlated with either acini count or TDLU span (Additional file 2: Table S2). Correlations observed for IGF and TDLU measures with mammographic density among pre- and postmenopausal women were attenuated after removing the effects of age at biopsy and BMI (Table 2).

**Table 2:** Distribution of IGF levels and TDLU characteristics among women with benign breast disease, stratified by menopausal status

	All women (n = 228)				Premenopausal (n = 155)				Postmenopausal (n = 73)				
	Median	Range	Correlation with density, *r*	Adj. correlation with density, *r*	Median	Range	Correlation with density, *r*	Adj. correlation with density, *r*	Median	Range	Correlation with density, *r*	Adj. correlation with density, *r*	*P* value^a^
IGF measures													
IGF-I (ng/ml)	116.15	20.68–250.85	0.08	−0.11	119.4	55.69–250.85	0.0001	−0.11	110.87	20.68–213.43	−0.05	−0.09	0.001
IGFBP-3 (ng/ml)	3451.25	1165.0–4926.5	−0.22^**^	−0.14^*^	3419.5	1784.5–4926.5	−0.25^**^	−0.13	3467	1165.0–4878.5	−0.18	−0.12	0.44
IGF-I:IGFBP-3 Molar ratio	0.122	0.062–0.268	0.27^**^	−0.03	0.13	0.071–0.074	0.20^*^	−0.05	0.11	0.062–0.189	0.08	−0.01	0.0001
Lobular involution Measures													
TDLU count^b^	4	0–100	0.22^*^	0.02	5	0–100	0.21*	0.005	3	0–79	0.14	0.02	0.16
Median TDLU span, μm	266	78–1030	0.17*	0.08	292	78–1030	0.14	0.08	202	83–568	−0.17	−0.001	0.0001
Median category of acini counts per TDLU	1	1–6	0.09	0.03	1	1–6	0.11	0.04	1	1–5	−0.10	−0.008	0.35

Correlation between insulin-like growth factor (*IGF*) and involution measures (continuous) with percent volumetric mammographic density was calculated using Spearman partial rank-order correlation. Adjusted (*Adj.*) correlation was calculated after removing the effects of age and body mass index. *P <0.05, **P <0.001. ^a^ *P* values comparing levels of IGFs for premenopausal and postmenopausal women were calculated using Kruskal–Wallis rank test. ^b^Terminal duct lobular unit (*TDLU*) count refers to numbers of TDLUs per unit of tissue area. *r* Spearman rho, *IGFBP-3* insulin-like growth factor binding protein-3

Among all women combined, IGF-I levels in the highest tertile versus the lowest were associated with higher TDLU counts in fully adjusted models (RR_T3vsT1_ = 1.31, 95 % CI = 1.18–1.45; *P-*trend <0.0001; Table 3). IGFBP-3 levels in the highest tertile versus the lowest were associated with lower TDLU counts (RR_T3vsT1_ = 0.88, 95 % CI = 0.80–0.96; *P-*trend = 0.009). IGF-I:IGFBP-3 ratio levels in the highest tertile versus the lowest were associated with higher TDLU counts (RR_T3vsT1_ = 1.84, 95 % CI = 1.65–2.06; *P-*trend <0.0001; Table 3). Results for postmenopausal women were similar to those for all women, although effects for IGFBP-3 (RR_T3vsT1_ = 0.56, 95 % CI = 0.45–0.70; *P-*trend <0.0001) and the IGF-I:IGFBP-3 ratio (RR_T3vsT1_ = 2.78, 95 % CI = 2.14–3.62; *P-*trend <0.0001; Table 3) were stronger. In contrast, only IGF-I:IGFBP-3 ratio levels in premenopausal women were associated with higher TDLU counts (RR_T3vsT1_ = 1.17, 95 % CI = 1.04–1.32; *P-*trend = 0.01). IGF-I and IGFBP-3 and their ratio were not significantly related to TDLU span or number of acini per TDLU (Additional file 3: Table S3 and Additional file 4: Table S4).

**Table 3:** Associations between IGF levels and TDLU count among women with benign breast disease, overall and stratified by menopausal status

	All women		Premenopausal^a^		Postmenopausal^b^	
IGF measure^c^	Number	RR (95 % CI)	Number	RR (95 % CI)	Number	RR (95 % CI)
IGF-I						
Tertile 1 (ref.)	76	1	52	1	25	1
Tertile 2	76	1.12 (1.01–1.23)	52	0.95 (0.85–1.06)	24	1.02 (0.83–1.26)
Tertile 3	76	1.31 (1.18–1.45)	51	1.00 (0.90–1.13)	24	1.26 (1.06–1.50)
*P-*trend	<0.0001		0.89		0.009	
IGFBP-3						
Tertile 1 (ref.)	74	1	52	1	24	1
Tertile 2	76	1.03 (0.94–1.13)	52	0.94 (0.84–1.05)	24	0.57 (0.46–0.71)
Tertile 3	76	0.88 (0.80–0.96)	51	1.00 (0.90–1.11)	24	0.56 (0.45–0.70)
*P-*trend	0.009		0.99		<0.0001	
IGF-I:IGFBP-3 molar ratio						
Tertile 1 (ref.)	75	1	52	1	24	1
Tertile 2	76	1.44 (1.29–1.60)	52	1.14 (1.01–1.28)	24	1.69 (1.28–2.23)
Tertile 3	75	1.84 (1.65–2.06)	51	1.17 (1.04–1.32)	24	2.78 (2.14–3.62)
*P-*trend	<0.0001		0.01		<0.0001	

Terminal duct lobular unit (*TDLU*) count refers to numbers of TDLUs per unit of tissue area. Relative risk (*RR*) and 95 % CI were estimated using zero-inflated Poisson regression analysis. Analyses among all women were adjusted for covariates included in both the premenopausal and postmenopausal women. *P* value for trend (*P*-*trend*) was calculated using the Wald test. ^a^Fully adjusted models for premenopausal women included body mass index (BMI), age at first birth, percent fat on the tissue slide and age at biopsy. ^b^Fully adjusted models for postmenopausal women included covariates in premenopausal women in addition to age at menarche and an interaction term for BMI and percent fat on the tissue slide. Note: analyses of insulin-like growth factor binding protein-3 (*IGFBP-3*) and the molar ratio in postmenopausal women and all women combined were additionally adjusted for age at menarche. Two women, one premenopausal and one postmenopausal, were missing data on this risk factor and were not included. ^c^Tertiles (T) for all women: insulin-like growth factor (*IGF-I*) (T1, <103; T2, 103 to <128; T3, 128+ ng/ml); IGFBP-3 (T1, <3,110; T2, 3,110 to <3,677; T3, 3,677+ ng/ml); molar ratio (T1, <0.113; T2, 0.113 to <0.133; T3, 0.133+). Tertiles for premenopausal women: IGF-I (T1, <107; T2, 107 to <132.3; T3, 132.3+ ng/ml); IGFBP-3 (T1, <3,079; T2, 3,079 to <3,668; T3, 3,668+ ng/ml); molar ratio (T1, <0.119; T2, 0.119 to <0.140; T3, 0.140+). Tertiles for postmenopausal women: IGF-I (T1, <93; T2, 93 to <122; T3, 122+ ng/ml); IGFBP-3 (T1, <3,214; T2, 3,214 to <3,838; T3, 3,839+ ng/ml); molar ratio (T1, <0.102; T2, 0.102 to <0.1205; T3, 0.1205+).

Findings from sensitivity analyses restricted to premenopausal women with available hormone data (n = 86) were consistent with those observed among all premenopausal/perimenopausal women combined, such that those in the highest tertile of IGF-I:IGFBP-3 ratio levels had higher TDLU counts (data not shown). In addition, patterns of association between IGF-I or IGFBP-3 and TDLU counts were similar to and somewhat stronger than those reported in Table 3, following mutual adjustment for IGFBP-3 and IGF-I, respectively (Additional file 5: Table S5).

### Relationships between IGF-I or IGFBP-3, and TDLU count, stratified by mammographic density

**Fig. 1:**
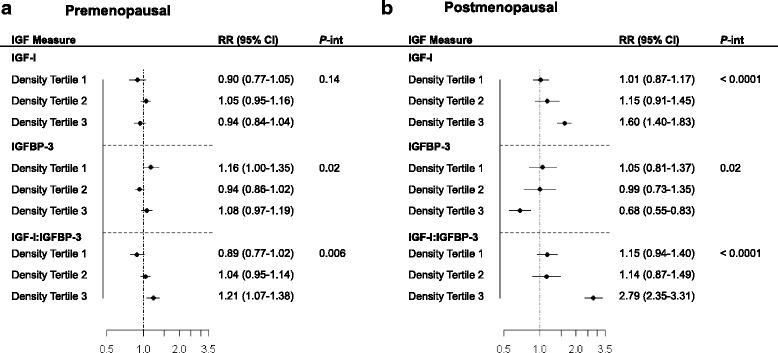
Association between insulin-like growth factor (*IGF*) levels and terminal duct lobular unit (TDLU) count among women with benign breast disease stratified by mammographic density. The association between levels of IGF proteins including IGF-I, IGF binding protein-3 (*IGFBP-3*) and the IGF-I:IGFBP-3 molar ratio, and TDLU count (modeled continuously) stratified by mammographic density are shown for **a** premenopausal women adjusted for age at biopsy, body mass index (BMI), age at first birth and percent fat on the tissue slide; and **b** postmenopausal women adjusted for age at biopsy, BMI, age at first birth and percent fat on the tissue slide. Analysis of IGFBP-3 and the molar ratio, but not IGF-I, in postmenopausal women was further adjusted for age at menarche, with the exception of the stratum for the highest tertile of mammographic density. Relative risk (*RR*) and 95 % confidence intervals (*CI*) were estimated using zero-inflated Poisson regression analysis, the outcome for the analysis was TDLU count (modeled continuously) and the independent variable was tertile of IGF-measure. These analyses were stratified by tertile of percent volumetric mammographic density; density tertiles were as follows: premenopausal: T1, <30.5 %; T2, 30.5 to <53 %; T3 ≥ 53.3 % and postmenopausal: T1, <22 %; T2, 22 to <33.3 %; T3, ≥33.3 %. *P-int* *=* *P* value for interaction

IGFBP-3 was inversely correlated with mammographic density and the IGF-I:IGFBP-3 ratio was positively correlated with mammographic density, but only significantly so among premenopausal women (*r* = –0.22 and *r* = 0.27, respectively; *P* <0.05; Table 2). Similarly, TDLU count was positively correlated with mammographic density, particularly among premenopausal women (Table 2). These associations were attenuated after adjustment for age and BMI (Table 2). Examination of the relationship between TDLU count and circulating IGF levels by tertiles of mammographic density revealed differences in the strength of the association for women across the density spectrum. As shown in Fig. 1a, among premenopausal women a positive association between the IGF-I:IGFBP-3 ratio and TDLU count was observed for women in the highest tertile of mammographic density (*P*-int = 0.006). For postmenopausal women (Fig. 1b), mammographic density modified all IGF associations with TDLU count; associations were strongest among those with higher mammographic density (IGF-I, *P*-int <0.0001; IGFBP-3, *P*-int = 0.02; and IGF-I:IGFBP-3 ratio, *P*-int <0.0001). Patterns of association were similar, though stronger, following mutual adjustment for IGFBP-3 and IGF-I (Additional file 6: Figure S1).

## Discussion

Our study demonstrates that elevated IGF-I:IGFBP-3 ratios among women with biopsy-proven benign breast disease were associated with higher TDLU counts, a measure of decreased TDLU involution, which has been linked to increased breast cancer risk. The associations between the IGF-I:IGFBP-3 ratio and TDLU count were strongest among postmenopausal women who had high mammographic density. In conjunction with mechanistic studies performed in animal models, our results suggest that studies assessing IGF levels and TDLU involution as markers of breast cancer risk are warranted. In addition, our findings further highlight the influence of the growth factor milieu on breast tissue architecture and pliability in the carcinogenic process.

Relationships between IGF-I, IGFBP-3 and the ratio of the two with TDLU measures differed by menopausal status. TDLU count was associated with IGF-I, IGFBP-3 and their ratio in postmenopausal women; however, only the IGF-I:IGFBP-3 ratio was associated with TDLU count in premenopausal women. The degree to which circulating hormones and growth factors interact within the tissue microenvironment [32–34] may partially account for the observed differences in associations by menopausal status. Moreover, as TDLU involution typically increases with age [6] and IGF-I decreases with age [35], future, larger studies of pre-, peri- and postmenopausal women will be important for gaining further insight into how the IGF system exerts its effects on breast architecture and tumor development across the life course.

Consistent with our findings, prior work suggests that increased mammographic density is associated with less TDLU involution [14, 16], and in multivariable risk models, having no or partial lobular involution has been associated with increased breast cancer risk, independent of mammographic density [1]. Several studies have also linked high levels of circulating IGF-I and low levels of IGFBP-3 to elevated mammographic density among pre- [26, 36, 37] and postmenopausal [38] women. However, most studies have been null among postmenopausal women ([39–41]; reviewed in [42]). Our findings are consistent with this literature in that we observed correlations between IGFs and mammographic density only among premenopausal women in our study population. Notably, we found that mammographic density modified associations with TDLU count for the IGF-I:IGFBP-3 ratio in premenopausal women and for all IGF measures in postmenopausal women, such that associations were strongest among those with higher mammographic density.

**Fig. 2:**
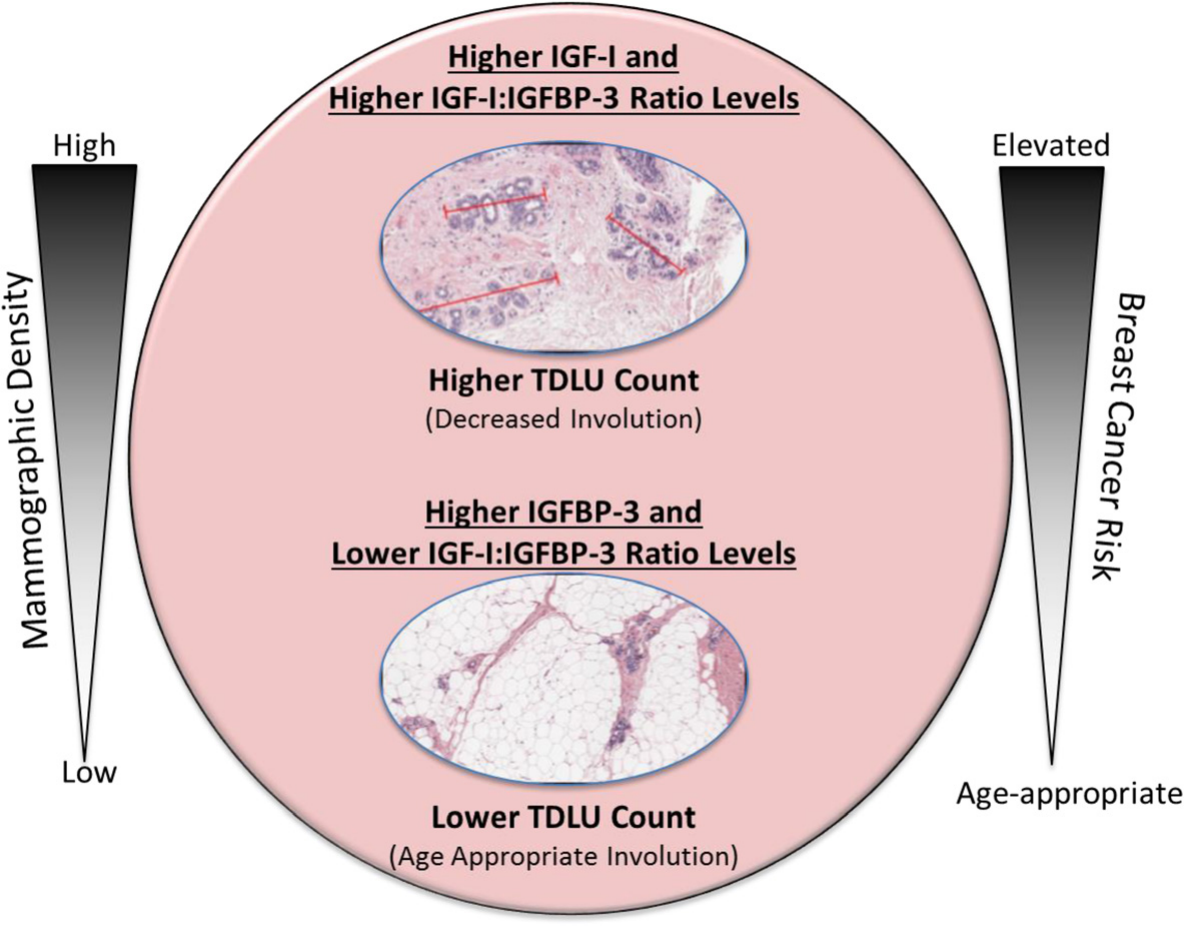
Hypothesized association between insulin-like growth factor (*IGF*) levels, terminal duct lobular unit (*TDLU*) involution, mammographic density and breast cancer risk. Circulating IGF levels in the breast may influence the parenchymal composition and organization leading to alterations in the surrounding epithelial tissue whereby higher levels of IGF-I and IGF-I:insulin-like growth factor binding protein-3 (*IGFBP-3*) ratio result in higher TDLU counts, decreased TDLU involution and presumably increased breast cancer risk. On the other hand, higher IGFBP-3 and lower circulating levels of IGF-I:IGFBP-3 ratio may permit the involution process to proceed, resulting in age-appropriate TDLU involution and decreased risk of breast cancer. Higher mammographic density, a strong breast cancer risk factor, may modify the IGF/involution relationship such that IGF may exert its greatest influence on TDLU involution among women with the highest mammographic density. *Red lines* (*top* H&E image) (higher TDLU count) indicate the span of the TDLU as measured by an electronic ruler

Assessing whether adding IGF levels, TDLU involution and mammographic density enhance prediction of developing breast cancer among women with biopsy-proven benign breast disease may be useful. We hypothesize a parallel role for the IGF system among women, whereby this pathway and its related networks influence breast cancer risk by altering parenchymal composition, organization and function (Fig. 2). Interestingly, local expression of IGF-I in the mammary gland of transgenic mice was found sufficient to stimulate increased milk yield, providing additional evidence in support of this hypothesis [9]. Further, given that mammographic density is a strong breast cancer risk factor reflecting the fibroglandular content of the breast [15, 42], the IGF system may link the associations between mammographic density, TDLU involution and breast cancer risk (Fig. 2).

To our knowledge, we are the first to demonstrate an association between circulating IGF levels and TDLU count as a measure of lobular involution. Rice and colleagues found that higher IGF-I levels and IGF-I:IGFBP-3 ratios were associated with less involuted TDLU categories, based on visual evaluation, which in turn was related to increased risk of breast cancer among women with benign breast disease [5]. In a transgenic mouse model, the IGF system has been shown to be effective in inhibiting involution of the lactating mammary gland [43]. These results, along with the current report, suggest a role for IGFs in TDLU involution and breast cancer risk.

We did not identify any significant associations between IGFs and TDLU span or acini count per TDLU, two additional measures of TDLU involution. TDLU span and acini count are highly correlated, whereas TDLU count is weakly correlated with either measure. Thus, TDLU span and acini count may reflect biological processes distinct from TDLU counts. Alternatively, associations may vary with TDLU metrics secondary to measurement and analysis. In prior analyses [6, 25], we have found that risk factor associations vary by the three TDLU metrics that we analyzed. One hypothesis is that TDLU span and acini content may vary more over time, whereas total loss of TDLUs could represent a relatively irreversible alteration. In addition, in highly involuted breast tissue, TDLUs may be absent, precluding assessment of span or acinar content in these individuals. Using ZIP regression allowed us to account for the number of samples with zero TLDU counts, which is important as such specimens may represent a profound degree of TDLU involution, although sampling represents another possible explanation in some women.

A major strength of our study was the utilization of highly reproducible measures of TDLU involution. A recent study found that TDLU counts were positively associated with number of live births and family history of breast cancer, and inversely associated with older age at menarche among postmenopausal women [6]. Also, analyses within the Mayo Benign Breast Disease Cohort found a stepwise increase in breast cancer risk with increasing numbers of acini per lobule [4]. Findings from the Mayo Cohort noting strong intra-woman concordance of subjectively assessed involution, measured in four quadrants of both breasts of 15 women who had undergone prophylactic mastectomy [44], also support the notion that measures of TDLU involution reflect a global process occurring throughout the breast and are general markers of breast cancer risk [3, 16]. These data in concert with our findings provide support for developing improved metrics of TDLU involution and its assessment as a potential intermediate measure of breast cancer risk.

A limitation of our study is that circulating levels of growth hormones were used, and analysis of tissue levels might prove more informative. In an analysis of healthy women, plasma and breast tissue levels of IGF-I and IGFBP-3 were not associated; however, breast cancer risk factors associated with plasma IGF-I and IGFBP-3 were similarly associated with breast concentrations of IGF-I and IGFBP-3 [45]. Another limitation of our study is that we were unable to stratify our data by menstrual cycle phase because of small numbers within each category, and thus, could not adjust for potential fluctuations in IGF-I levels over the menstrual cycle [46]. However, TDLU structure and mammographic density are unlikely to vary greatly during menstrual cycles [6, 47]. Similarly, we were unable to model benign diagnoses more specifically because of limited sample size. These limitations would likely have biased our findings toward the null. Finally, given the variation in IGF-I and IGFBP-3 levels by racial/ethnic groups [48–51], our findings may not apply to non-white women, as white women comprised nearly all participants in this study population.

## Conclusion

In summary, we demonstrated that circulating levels of serum IGF-I and IGFBP-3 are associated with TDLU count among women with benign breast disease, and that the strongest associations are found among women who have high mammographic density. Our findings contribute to the body of knowledge that supports the evaluation of TDLU involution and mammographic density as possible intermediate endpoints in breast carcinogenesis. Expanding this analysis to a study of breast cancer risk may further contribute to an improved understanding of the role of TDLU involution, or lack thereof, in the mammary carcinogenic process, which could have value for improving risk prediction and prevention.

## Additional files

Additional file 1: Table S1. Radiologic and pathologic characteristics of women in the BREAST Stamp Project with benign breast disease, stratified by menopausal status. (PDF 46 kb) Additional file 2: Table S2. Correlations between terminal duct lobular unit (TDLU) measures. (PDF 74 kb) Additional file 3: Table S3. Associations between IGF levels and median TDLU span among women with benign breast disease, overall and stratified by menopausal status. (DOC 48 kb) Additional file 4: Table S4. Associations between IGF levels and median category of acini count per TDLU among women with benign breast disease, overall and stratified by menopausal status. (DOC 46 kb) Additional file 5: Table S5. Associations of IGF levels and TDLU count among women with benign breast disease with mutual adjustment for IGFBP-3 and IGF-I, overall and stratified by menopausal status. (DOC 44 kb) Additional file 6: Figure S1. Association between IGF levels and TDLU count among women with benign breast disease with mutual adjustment for IGFBP-3 and IGF-I, stratified by mammographic density. (DOC 157 kb)

## Abbreviations

BMI: body mass index
BREAST: Breast Radiology Evaluation and Study of Tissues
CI: confidence interval
CV: coefficient of variation
H&E: hematoxylin and eosin
IGFBP-3: insulin-like growth factor binding protein-3
IGF-I: insulin-like growth factor-I
OR: odds ratio
*P*-int: *P* value for interaction
RR: relative risk
SXA: single x-ray absorptiometry
TDLU: terminal duct lobular unit
UVM: University of Vermont
ZIP: zero-inflated Poisson regression
