# Supplementary material for: Circulating insulin-like growth factor-I, insulin-like growth factor binding protein-3 and terminal duct lobular unit involution of the breast: a cross-sectional study of women with benign breast disease
Source: Breast Cancer Res. 2016 Feb 18;18:24. doi: 10.1186/s13058-016-0678-4 (PMC4758090; doi:10.1186/s13058-016-0678-4)
Supplement: Additional file 1: Table S1. — Radiologic and pathologic characteristics of women in the BREAST Stamp Project with benign breast disease, stratified by menopausal status. (PDF 46 kb) [file 13058_2016_678_MOESM1_ESM.pdf]

**Table S1.** Radiologic and pathologic characteristics of women in the BREAST Stamp Project with benign breast disease, stratified by menopausal status

| | Premenopausal | | Postmenopausal | |
|---|---|---|---|---|
| | N = 155 | | N = 73 | |
| **Characteristic** | **N** | **%** | **N** | **%** |
| **Biopsy type[a]** | | | | |
| Ultrasound-guided | 88 | 56.8 | 30 | 41.1 |
| Stereotactic-guided | 67 | 43.2 | 43 | 58.9 |
| **Biopsy laterality** | | | | |
| Left | 78 | 50.3 | 37 | 50.7 |
| Right | 77 | 49.7 | 36 | 49.3 |
| **Pathologic diagnosis** | | | | |
| Benign | 66 | 42.6 | 29 | 39.7 |
| Proliferative | 79 | 51.0 | 33 | 45.2 |
| Proliferative with atypia | 10 | 6.4 | 11 | 15.1 |
| **Percent fat on H&E slide,[b] by biopsy type** | | | | |
| Ultrasound-guided | | | | |
| ≤ 30 | 40 | 47.6 | 17 | 56.7 |
| > 30 - 60 | 18 | 21.4 | 7 | 23.3 |
| > 60 - 100 | 26 | 31.0 | 6 | 20.0 |
| Stereotactic-guided | | | | |
| ≤ 30 | 25 | 37.3 | 18 | 42.9 |
| > 30 - 60 | 15 | 22.4 | 12 | 28.6 |
| > 60 - 100 | 27 | 40.3 | 12 | 28.6 |
| | **Mean** | **95% CI** | **Mean** | **95% CI** |
| **Volumetric mammographic density measures[c]:** | | | | |
| Percent fibroglandular volume (%) | 43.2 | 39.65 - 46.79 | 31.3 | 26.78 - 35.62 |
| Absolute fibroglandular volume ($cm^3$) | 204.4 | 187.78 - 221.99 | 186.6 | 161.67 - 204.47 |
| **Area mammographic density measures[c]:** | | | | |
| Percent dense area (%) | 31.5 | 28.29 - 34.57 | 20.9 | 16.70 - 25.38 |
| Absolute dense area ($cm^2$) | 38.5 | 34.29 - 42.97 | 28.8 | 23.52 - 34.92 |

Abbreviations: H&E = hematoxylin and eosin
[a]There were also participants in the BREAST Stamp Project who received an either MRI-guided biopsy or surgical excision, these individuals were not included in our analytic sample. .
[b]Percent fat present in the histological specimen selected for assessment of terminal duct lobular unit (TDLU) involution.
[c]Density measures are from the ipsilateral breast on which the biopsy was performed and TDLU measures assessed.
