# Supplementary material for: Circulating insulin-like growth factor-I, insulin-like growth factor binding protein-3 and terminal duct lobular unit involution of the breast: a cross-sectional study of women with benign breast disease
Source: Breast Cancer Res. 2016 Feb 18;18:24. doi: 10.1186/s13058-016-0678-4 (PMC4758090; doi:10.1186/s13058-016-0678-4)
Supplement: Additional file 2: Table S2. — Correlations between terminal duct lobular unit (TDLU) measures. (PDF 74 kb) [file 13058_2016_678_MOESM2_ESM.pdf]

Table S2: Correlations between TDLU measures

**TDLU Measure**	**Median category of acini counts per TDLU, *r***	**Median TDLU span (µm), r**
**All women**		
TDLU count [1]	0.09	0.0005
Median TDLU span, µm	0.62*	~
**Postmenopausal Women**		
**TDLU Measure**	**Median category of acini counts per TDLU, *r***	**Median TDLU span (µm), r**
TDLU count [1]	0.10	0.03
Median TDLU span, µm	0.68*	~
**Premenopausal women**		
**TDLU Measure**	**Median category of acini counts per TDLU, *r***	**Median TDLU span (µm), r**
TDLU count [1]	0.05	-0.18
Median TDLU span, µm	0.55*	~

Abbreviations: TDLU = terminal duct lobular unit, *r* = Spearman rho
Correlations between TDLU measures were assessed by Spearman rank correlation, *P <0.001.
[1]TDLU count refers to numbers of TDLUs per unit of tissue area.
