# Supplementary material for: Circulating insulin-like growth factor-I, insulin-like growth factor binding protein-3 and terminal duct lobular unit involution of the breast: a cross-sectional study of women with benign breast disease
Source: Breast Cancer Res. 2016 Feb 18;18:24. doi: 10.1186/s13058-016-0678-4 (PMC4758090; doi:10.1186/s13058-016-0678-4)
Supplement: Additional file 3: Table S3. — Associations between IGF levels and median TDLU span among women with benign breast disease, overall and stratified by menopausal status. (DOC 48 kb) [file 13058_2016_678_MOESM3_ESM.doc]

| **Table S3.** Associations between IGF levels and median TDLU span* among women with benign breast disease, overall and stratified by menopausal status | | | | | | |
| --- | --- | --- | --- | --- | --- | --- |
|  | **All women** | | **Premenopausal^a^** | | **Postmenopausal^b^** | |
|  |  | **Fully adjusted Model** |  | **Fully adjusted Model** |  | **Fully adjusted Model** |
| **IGF measure^c^** | **N** | **OR (95% CI)** | **N** | **OR (95% CI)** | **N** | **OR (95% CI)** |
| **IGF-I** |  |  |  |  |  |  |
| Tertile 1 (ref.) | 49 | 1.00 | 37 | 1.00 | 16 | 1.00 |
| Tertile 2 | 56 | 1.21 (0.58-2.55) | 38 | 1.03 (0.45-2.38) | 15 | 1.54 (0.36-6.47) |
| Tertile 3 | 51 | 1.17 (0.54-2.52) | 37 | 0.91 (0.40-2.06) | 14 | 1.85 (0.39-8.74) |
| *P-trend* |  | *0.70* |  | *0.82* |  | *0.43* |
| **IGFBP-3** |  |  |  |  |  |  |
| Tertile 1 (ref.) | 54 | 1.00 | 39 | 1.00 | 17 | 1.00 |
| Tertile 2 | 52 | 1.00 (0.48-2.07) | 39 | 0.98 (0.44-2.20) | 12 | 0.73 (0.13-4.21) |
| Tertile 3 | 50 | 1.30 (0.62-2.72) | 34 | 1.51 (0.65-3.51) | 16 | 0.78 (0.19-3.20) |
| *P-trend* |  | *0.49* |  | *0.35* |  | *0.74* |
| **IGF-I:IGFBP-3 Molar Ratio** |  |  |  |  |  |  |
| Tertile 1 (ref.) | 49 | 1.00 | 33 | 1.00 | 16 | 1.00 |
| Tertile 2 | 49 | 0.68 (0.30-1.53) | 38 | 0.76 (0.31-1.82) | 16 | 1.65 (0.34-7.99) |
| Tertile 3 | 58 | 0.88 (0.38-2.03) | 41 | 0.89 (0.38-2.09) | 13 | 1.90 (0.34-10.71) |
| *P-trend* |  | *0.83* |  | *0.85* |  | *0.46* |
| Abbreviations: TDLU = terminal duct lobular unit; IGF = insulin like growth factor; IGFBP-3 = insulin like growth factor binding protein -3; OR = odds ratio; CI = confidence interval; ref. = reference | | | | | | |
| OR and 95% CI were estimated using an ordinal logistic regression model among women with TDLUs. P-value for trend (P-trend) was calculated using Wald tests. | | | | | | |
| **^a^**Models for premenopausal women were adjusted age at biopsy . | | | | | | |
| **^b^**Models for postmenopausal women were adjusted for age at biopsy, body mass index (BMI), and years since menopause. Analyses among all women were adjusted for covariates included in both premenopausal and postmenopausal fully adjusted models.  ^c^ Tertiles all women: IGF-I (T1: <103; T2: 103-<128; T3: 128+ ng/ml); IGFBP-3 (T1: <3110; Tert2: 3110-<3677; Tert3: 3677+ ng/ml); Molar Ratio (T1: <0.113; T2: 0.113-<0.133; T3: 0.133+).  Tertiles premenopausal women: IGF-I (T1: <107; T2: 107-<132.3; T3: 132.3+ ng/ml); IGFBP-3 (T1: <3079; T2: 3079-<3668; T3: 3668+ ng/ml); Molar Ratio (T1: <0.119; T2: 0.119-<0.140; T3: 0.140+).  Tertiles postmenopausal women: IGF-I (T1: <93; T2: 93-<122; T3: 122+ ng/ml); IGFBP-3 (T1: <3214; T2: 3214-<3838; T3: 3839+ ng/ml); Molar Ratio (T1: <0.102; T2: 0.102-<0.1205; T3: 0.1205+).  *The dependent variable, TDLU span, was modeled in tertiles: premenopausal (T1:<258; T2: 258-<339; T3: 339+ microns) and postmenopausal (T1: <79; T2: 79-<232; T3: 232+ microns). | | | | | | |
