# Supplementary material for: Circulating insulin-like growth factor-I, insulin-like growth factor binding protein-3 and terminal duct lobular unit involution of the breast: a cross-sectional study of women with benign breast disease
Source: Breast Cancer Res. 2016 Feb 18;18:24. doi: 10.1186/s13058-016-0678-4 (PMC4758090; doi:10.1186/s13058-016-0678-4)
Supplement: Additional file 4: Table S4. — Associations between IGF levels and median category of acini count per TDLU among women with benign breast disease, overall and stratified by menopausal status. (DOC 46 kb) [file 13058_2016_678_MOESM4_ESM.doc]

| **Table S4.** Associations between IGF levels and median category of acini count* per TDLU among women with benign breast disease, overall and stratified by menopausal status | | | | | | |
| --- | --- | --- | --- | --- | --- | --- |
| **IGF measure** | **All women^a^** | | **Premenopausal^a^** | | **Postmenopausal^a^** | |
|  | **N** | **OR (95% CI)** | **N** | **OR (95% CI)** | **N** | **OR (95% CI)** |
| **IGF-I** |  |  |  |  |  |  |
| Tertile 1 (ref.) | 52 | 1.00 | 38 | 1.00 | 17 | 1.00 |
| Tertile 2 | 59 | 1.61 (0.76-3.38) | 40 | 0.82 (0.34-1.96) | 17 | 0.53 (0.13-2.19) |
| Tertile 3 | 55 | 1.54 (0.71-3.33) | 38 | 0.96 (0.41-2.25) | 16 | 1.88 (0.48-7.32) |
| *P-trend* |  | *0.28* |  | *0.94* |  | *0.35* |
| **IGFBP-3** |  |  |  |  |  |  |
| Tertile 1 (ref.) | 57 | 1.00 | 40 | 1.00 | 18 | 1.00 |
| Tertile 2 | 54 | 0.80 (0.38-1.68) | 40 | 0.87 (0.37-2.07) | 13 | 0.38 (0.08-1.92) |
| Tertile 3 | 55 | 1.31 (0.64-2.67) | 36 | 1.11 (0.47-2.64) | 19 | 1.31 (0.36-4.79) |
| *P-trend* |  | *0.45* |  | *0.81* |  | *0.62* |
| **IGF-I:IGFBP-3 Molar Ratio** |  |  |  |  |  |  |
| Tertile 1 (ref.) | 53 | 1.00 | 34 | 1.00 | 17 | 1.00 |
| Tertile 2 | 51 | 0.77 (0.36-1.65) | 39 | 0.91 (0.37-2.22) | 18 | 0.88 (0.23-3.43) |
| Tertile 3 | 62 | 0.90 (0.41-2.01) | 43 | 1.15 (0.48-2.78) | 15 | 1.06 (0.26-4.29) |
| *P-trend* |  | *0.83* |  | *0.73* |  | *0.93* |
| Abbreviations: TDLU = terminal duct lobular unit; IGF = insulin like growth factor; IGFBP-3 = insulin like growth factor binding protein -3; OR = odds ratio; CI = confidence interval; ref. = reference | | | | | | |
| OR and 95% CI were estimated using an ordinal logistic regression model among women with TDLUs. P-value for trend (P-trend) was calculated using Wald tests. | | | | | | |
| **^a^**Models shown are adjusted for age at biopsy only, as there were no significant confounders in either the premenopausal or postmenopausal women. | | | | | | |
| *The dependent variable, category of acini count per TDLU (categories: 1: ≤10, 2: 11-20, 3: 21-30, 4: 31-50, 5: 51-100, and 6: >100), were modeled in tertiles: premenopausal (T1:<1.5; T2: 1.5-<2.5; T3: 2.5+) and postmenopausal (T1:<1.5; T2: 1.5-<2; T3: 2+). | | | | | | |
