# Supplementary material for: Circulating insulin-like growth factor-I, insulin-like growth factor binding protein-3 and terminal duct lobular unit involution of the breast: a cross-sectional study of women with benign breast disease
Source: Breast Cancer Res. 2016 Feb 18;18:24. doi: 10.1186/s13058-016-0678-4 (PMC4758090; doi:10.1186/s13058-016-0678-4)
Supplement: Additional file 5: Table S5. — Associations of IGF levels and TDLU count among women with benign breast disease with mutual adjustment for IGFBP-3 and IGF-I, overall and stratified by menopausal status. (DOC 44 kb) [file 13058_2016_678_MOESM5_ESM.doc]

| **Table S5.** Associations between IGF levels and TDLU count* among women with benign breast disease with mutual adjustment for IGFBP-3 and IGF-I, overall and stratified by menopausal status | | | | | | |
| --- | --- | --- | --- | --- | --- | --- |
|  |  | **All Women** |  | **Premenopausal^a^** |  | **Postmenopausal^b^** |
| **IGF measure^c^** | **N** | **RR (95% CI)** | **N** | **RR (95% CI)** | **N** | **RR (95% CI)** |
| **IGF-I (adj for IGFBP-3)** |  |  |  |  |  |  |
| Tertile 1 (ref.) | 76 | 1 | 52 | 1 | 24 | 1 |
| Tertile 2 | 76 | 1.18 (1.06-1.31) | 52 | 0.97 (0.86-1.09) | 22 | 1.49 (1.14-1.95) |
| Tertile 3 | 76 | 1.47 (1.31-1.65) | 51 | 1.02 (0.90-1.16) | 23 | 3.73 (2.81-4.95) |
| *P-trend* |  | *< 0.0001* |  | *0.68* |  | *< 0.0001* |
| **IGFBP-3 (adj for IGF-I)** |  |  |  |  |  |  |
| Tertile 1 (ref.) | 74 | 1 | 52 | 1 | 22 | 1 |
| Tertile 2 | 76 | 0.92 (0.83-1.02) | 52 | 0.94 (0.83-1.07) | 22 | 0.29 (0.21-0.40) |
| Tertile 3 | 76 | 0.76 (0.69-0.84) | 51 | 0.99 (0.88-1.11) | 24 | 0.21 (0.15-0.28) |
| *P-trend* |  | *< 0.0001* |  | *0.94* |  | *< 0.0001* |
| Abbreviations: TDLU = terminal duct lobular unit; IGF = insulin like growth factor; IGFBP-3 = insulin like growth factor binding protein -3; RR = relative risk; CI = confidence interval | | | | | | |
| *TDLU count refers to numbers of TDLUs per unit of tissue area. | | | | | | |
| RR and 95% CI were estimated using zero-inflated Poisson regression analyses. Analyses among all women were adjusted for covariates included in both the premenopausal and postmenopausal women. *P*-value for trend *(P*-trend) was calculated using Wald tests. | | | | | | |
| **^a^**Fully adjusted models for premenopausal women included BMI, age at first birth, percent fat in the tissue slide and age at biopsy. Analyses relating IGF-I to TDLU count were additionally adjusted for IGFBP-3, and analyses relating IGFBP-3 to TDLU count were additionally adjusted for IGF-I. | | | | | | |
| **^b^**Fully adjusted models for postmenopausal women included covariates in premenopausal women in addition to age at menarche. | | | | | | |
| ^c^ Tertiles all women: IGF-I (T1: <103; T2: 103-<128; T3: 128+ ng/ml); IGFBP-3 (T1: <3110; Tert2: 3110-<3677; Tert3: 3677+ ng/ml); Molar Ratio (T1: <0.113; T2: 0.113-<0.133; T3: 0.133+). | | | | | | |
| Tertiles premenopausal women: IGF-I (T1: <107; T2: 107-<132.3; T3: 132.3+ ng/ml); IGFBP-3 (T1: <3079; T2: 3079-<3668; T3: 3668+ ng/ml); Molar Ratio (T1: <0.119; T2: 0.119-<0.140; T3: 0.140+). | | | | | | |
| Tertiles postmenopausal women: IGF-I (T1: <93; T2: 93-<122; T3: 122+ ng/ml); IGFBP-3 (T1: <3214; T2: 3214-<3838; T3: 3839+ ng/ml); Molar Ratio (T1: <0.102; T2: 0.102-<0.1205; T3: 0.1205+). | | | | | | |
