# Supplementary material for: Circulating insulin-like growth factor-I, insulin-like growth factor binding protein-3 and terminal duct lobular unit involution of the breast: a cross-sectional study of women with benign breast disease
Source: Breast Cancer Res. 2016 Feb 18;18:24. doi: 10.1186/s13058-016-0678-4 (PMC4758090; doi:10.1186/s13058-016-0678-4)
Supplement: Additional file 6: Figure S1. — Association between IGF levels and TDLU count among women with benign breast disease with mutual adjustment for IGFBP-3 and IGF-I, stratified by mammographic density. (DOC 157 kb) [file 13058_2016_678_MOESM6_ESM.doc]

**Supplementary Figure S1:**


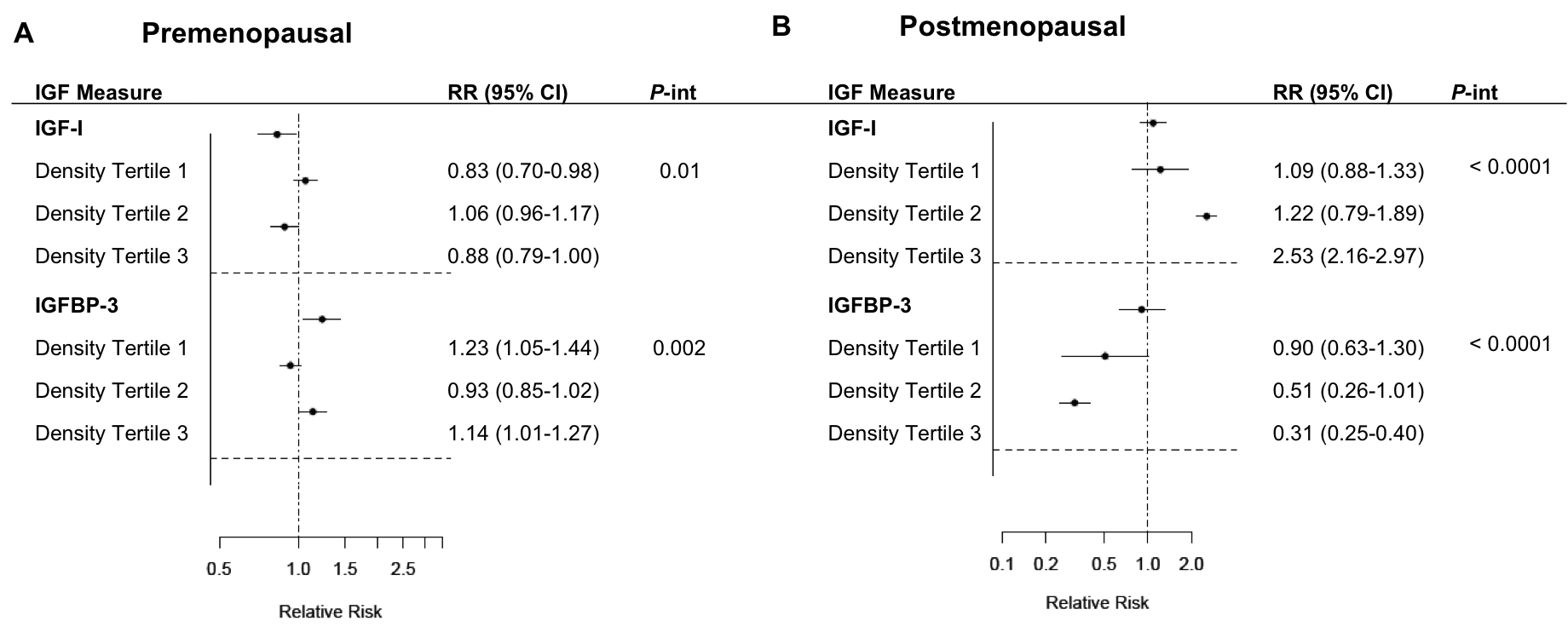


**Figure S1. Association between IGF levels and TDLU count among women with benign breast disease with mutual adjustment for IGFBP-3 and IGF-I, stratified by mammographic density.** The association between levels of insulin like growth factor (IGF) proteins including IGF-I, IGF binding protein-3 (IGFBP-3) and the IGF-I:IGFBP-3 molar ratio, and TDLU count (modeled continuously) stratified by mammographic density are shown for A) premenopausal women adjusted for age at biopsy, BMI, age at first birth and percent fat in the tissue slide; and B) postmenopausal women adjusted for age at biopsy, BMI, age at first birth, and percent fat in the tissue slide. Analyses of IGFBP-3, but not IGF-I, in postmenopausal women were further adjusted for age at menarche with the exception of the stratum for the highest mammographic density tertile. In addition, analyses relating IGF-I to TDLU count were additionally adjusted for IGFBP-3, and analyses relating IGFBP-3 to TDLU count were additionally adjusted for IGF-I. Relative risk (RR) and 95% confidence intervals (CI) were estimated using zero-inflated Poisson regression analyses, the outcome for the analyses was TDLU count (modeled continuously) and the independent variable was tertiles of IGF-measures. These analyses were stratified by tertiles of percent volumetric mammographic density; density tertiles were as follows: Premenopausal – T1 <30.5%; T2 30.5-<53.3%; T3 ≥53.3% and Postmenopausal - T1 <22%; T2 22-<33.3%; T3 ≥33.3%.
